# 3D-surface MALDI mass spectrometry imaging for visualising plant defensive cardiac glycosides in *Asclepias curassavica*

**DOI:** 10.1007/s00216-021-03177-y

**Published:** 2021-02-05

**Authors:** Domenic Dreisbach, Georg Petschenka, Bernhard Spengler, Dhaka R. Bhandari

**Affiliations:** 1 Institute for Inorganic and Analytical Chemistry, Justus Liebig University Giessen, Heinrich-Buff-Ring 17, 35392 Giessen, Germany; 2 Institute of Phytomedicine, University of Hohenheim, Otto-Sander-Straße 5, 70599 Stuttgart, Germany

**Keywords:** Mass spectrometry imaging, 3D-surface analysis, Plant chemical defence, Cardiac glycosides, *Asclepias curassavica*

## Abstract

Mass spectrometry–based imaging (MSI) has emerged as a promising method for spatial metabolomics in plant science. Several ionisation techniques have shown great potential for the spatially resolved analysis of metabolites in plant tissue. However, limitations in technology and methodology limited the molecular information for irregular 3D surfaces with resolutions on the micrometre scale. Here, we used atmospheric-pressure 3D-surface matrix-assisted laser desorption/ionisation mass spectrometry imaging (3D-surface MALDI MSI) to investigate plant chemical defence at the topographic molecular level for the model system *Asclepias curassavica*. Upon mechanical damage (simulating herbivore attacks) of native *A. curassavica* leaves, the surface of the leaves varies up to 700 μm, and cardiac glycosides (cardenolides) and other defence metabolites were exclusively detected in damaged leaf tissue but not in different regions of the same leaf. Our results indicated an increased latex flow rate towards the point of damage leading to an accumulation of defence substances in the affected area. While the concentration of cardiac glycosides showed no differences between 10 and 300 min after wounding, cardiac glycosides decreased after 24 h. The employed autofocusing AP-SMALDI MSI system provides a significant technological advancement for the visualisation of individual molecule species on irregular 3D surfaces such as native plant leaves. Our study demonstrates the enormous potential of this method in the field of plant science including primary metabolism and molecular mechanisms of plant responses to abiotic and biotic stress and symbiotic relationships.

**Supplementary Information:**

The online version contains supplementary material available at 10.1007/s00216-021-03177-y.

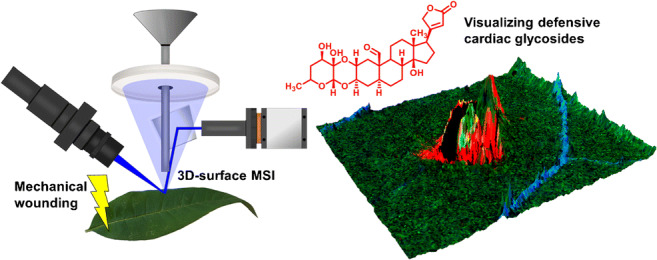

## Introduction

In the field of plant science, interest in techniques for imaging the spatial distribution of small molecules in plant tissue has grown rapidly. In this respect, mass spectrometry (MS)–based imaging (MSI) methods have emerged over the recent years. MSI provides label-free imaging and owing to its untargeted nature, not only the molecules of interest but also hundreds of other chemical species can be detected, identified and visualised simultaneously [1]. Several ambient ionisation techniques coexist and differ in their principles and properties. All of them have at least one distinct advantage over the others. Desorption electrospray ionisation (DESI) and laser ablation electrospray ionisation (LAESI) allow for investigation of objects and tissue sections without any sample preparation [2, 3]. However, the spatial resolution of DESI and LAESI (typically performed at 100 μm) does not reach the cellular level. MALDI, introduced in the mid-1990s [4], is by far the most extensively used MSI technique due to its broad applicability and high sensitivity at an excellent spatial resolution (5 to 100 μm for commercial instruments). The limitation of MALDI lies with the choice and application of the matrix substance, because these critical parameters determine analyte coverage, limits of detection, ionisation efficiency and spatial resolution.

Most MALDI MSI studies have focused on mammalian tissues. However, MSI of plant tissue is starting to catch up, and recent studies have proven the potential of MSI for the localisation of metabolites in plants, moving/advancing plant science beyond traditional botanical histochemistry. For instance, tissue-specific localisation of flavonoids in the rhizome of liquorice was visualised in the cork layer with 10-μm lateral resolution [5]. In rapeseed, more than 90 compounds were visualised, and plant tissues were visualised with 5 to 25-μm lateral resolution [6]. The MALDI MSI of primary and secondary metabolites in root sections of *Paeonia lactiflora* has been reported at 10- and 30-μm lateral resolution [7]. The cellular and subcellular distributions of amino acids, glycolipids and defence-related compounds in maize leaf sections were mapped at 5-μm pixel size with an oversampling method and by modifying the laser beam optics of a MALDI linear ion trap Orbitrap mass spectrometer [8]. A custom-built MALDI source coupled to an FTICR-MS enabled MALDI imaging of various metabolites in *Arabidopsis thaliana* sections at 10-μm lateral resolution [9]. Importantly, all of these studies have one aspect in common: the MSI experiments were conducted on planar sample surfaces (e.g. tissue sections). However, given the ubiquity of applications in a three-dimensional framework in the biological sciences, 3D MSI is emerging as a new frontier [10]. To this date, the most common approach for 3D MSI is based on serial tissue sections that are collected and imaged individually with 2D MSI techniques [10–12]. In the next step, a 3D image is reconstructed out of the generated 2D images making this method theoretically applicable to all MSI techniques. In contrast, creating 3D images via depth profiling (at atmospheric pressure) is only suitable for LAESI because several surface layers of a single tissue section are sputtered away, providing a submicrometre-depth resolution. For instance, several metabolites were visualised in radish leaves (*Raphanus sativus*) at 100-μm lateral resolution via a custom-built LAESI source coupled to an LTQ Orbitrap XL mass spectrometer [13]. The authors reported that the maximum z-distance between two particular ablation marks was 1.43 mm. The average diameters of ablation marks were 69 μm, demonstrating that the lateral resolution for 3D LAESI MSI is limited because a smaller laser beam diameter also means that less material will be ablated, resulting in lower ion yields which directly affects sensitivity. 3D-surface MALDI MSI, the most recent instrumental approach in AP-SMALDI MSI, overcomes sample height-related artefacts via a laser triangulation system [14]. This autofocusing AP-SMALDI MSI system enables the chemical analysis of irregular 3D sample surfaces with topographic aspect ratios (height to width) of up to 50 and providing spatial resolutions of ≤ 10 μm. For instance, lipid distributions on the surface of *Schistosoma mansoni*, a blood fluke with height variations up to 160 μm, was analysed with a spatial resolution of 5 μm [15].

Plants are challenged by a variety of abiotic and biotic stresses during their life cycle. Biotic stress, in particular, is represented by insect herbivores that depend on plants throughout their lifetime [16, 17]. Plant defensive traits against herbivores can be either direct or indirect. Direct defences involve the production of toxic secondary chemicals, which may either affect herbivores directly or impair their growth, development and the digestibility of the plant diet [18–20].

For instance, milkweed plants (*Asclepias spp*) produce toxic cardenolides that inhibit Na^+^/K^+^-ATPase, which is an essential ion carrier in animal cells [21, 22]. The general structure of cardenolides consists of a steroid molecule attached to a sugar unit and a five-membered lactone group, and up to 21 different cardenolides were identified in *Asclepias curassavica* via NMR, IR and LC-MS experiments [23].

In this work, we employ 3D-surface MALDI MSI for the spatially resolved analysis of plant chemical defence using *A. curassavica* as a model. The spatio-chemical results were obtained by analysing mechanically wounded (mimicking herbivore attack) *A. curassavica* leaf samples (height variations up to 700 μm). Numerous defence-related metabolites (including cardenolides) were exclusively detected and localised in damaged leaf tissue, demonstrating that latex is a vehicle for allocating chemical defences to the site of injury. Also, this study shows the capabilities and potential applicability of autofocusing MALDI mass spectrometry imaging regarding the analysis of plant samples in their native state and even on three-dimensional surfaces.

## Materials and methods

### Chemicals and plant samples

Trifluoroacetic acid (TFA), water (HPLC grade), acetone (HPLC grade) and 2,5-dihydroxybenzoic acid (DHB, 98% purity) were purchased from Sigma-Aldrich (Steinheim, Germany). *A. curassavica* plant samples were cultivated at the Institute for Insect Biotechnology, Justus Liebig University Giessen, Germany.

### Sample preparation for 3D-surface MALDI imaging

Mechanical wounding to mimic insect herbivory was carried out by using a sterile needle. The needle had a uniform diameter and was pulled in one direction perpendicular through the plant leaf of an intact plant. We made sure that no latex was spread from the needle to the leaf surface. After certain time intervals (10 min to 24 h depending on the experiments), the leaf was harvested for direct imaging measurements. For imaging experiments, samples were glued onto a MALDI target plate using double-sided duct tape. No washing steps were applied before matrix application. A solution of 30 mg/ml 2.5-dihydroxybenzoic acid in acetone/water (0.2% TFA) 1:1 v/v was freshly prepared for each matrix application. A volume of 100-μl DHB matrix solution was sprayed onto the sample with a flow rate of 10 μl/min and a rotation of 500 rpm using an ultrafine pneumatic sprayer system (SMALDIPrep, TransMIT GmbH, Giessen, Germany). 3D optical images of the sample surfaces were obtained with a Keyence VHX-5000 digital microscope (Keyence Deutschland GmbH, Neu-Isenburg, Germany) equipped with a VH-Z250R objective lens.

### Instrumentation for 3D-surface MALDI imaging

All measurements were performed using an autofocusing AP-SMALDI5 AF high-resolution MALDI imaging ion source (TransMIT GmbH, Giessen, Germany), which was operated at atmospheric pressure and coupled to a Q Exactive HF Orbitrap mass spectrometer (Thermo Fisher Scientific, Bremen, Germany). For desorption/ionisation, a diode-pumped solid-state laser at 343-nm wavelength, operating at 100 Hz and focused perpendicular to the sample to an effective ablation spot diameter of 5 μm, was used. The autofocusing system consists of a continuous-wave laser which irradiates the sample at an angle of 35° relative to the transfer capillary axis of the mass spectrometer. This system enabled us to keep the desorption/ionisation laser focus, fluence and ablation spot size constant across sample height differences (such as leaf veins, wounded plant tissue or the naturally uneven surface of an *A. curassavica* leaf) by adjusting the sample stage position according to the sample height profile for each measurement spot. For each mass spectrum, ions of 50 laser pulses were accumulated in the C-trap before being transferred into the Orbitrap mass analyser. The autofocusing AP-SMALDI ion source was operated using the SMALDIControl software package (TransMIT GmbH, Giessen, Germany). The samples were scanned with 20- to 45-μm step size. The step size of the xyz-sample stage was set to the desired pixel size. The mass spectrometer was operated in positive-ion mode in a mass-to-charge (*m*/*z*) range of 250 to 1000 at a mass resolution of 240,000 at *m*/*z* 200. Internal lock-mass calibration was performed by using a mass value of a DHB matrix cluster ion ([5DHB-4H_2_O + NH_4_]^+^, *m*/*z* 716.12461), resulting in a mass accuracy of better than 2 ppm root mean square error (RMSE). The scan speed for the pixel-wise autofocusing AP-SMALDI measurements was 1.6 s per pixel. The ion injection time was set to 500 ms. The S-lens level was set to 100 arbitrary units, and the capillary temperature was 250 °C.

### Sample preparation for HPLC-MS/MS analysis

Mechanical wounding was carried out by using the same procedure as for the imaging experiments. After 120 min, injured and intact leaf samples were harvested, and uniform leaf discs of the injured area and the corresponding area of the intact leaf were obtained using a puncher (see Supplementary Information (ESM) Fig. S1). Each leaf disc was freeze-dried and ground to a fine powder with the help of mortar and pestle. For every biological replicate, 5 mg of each powder was extracted with 2 ml of absolute methanol and incubated at 50 °C for 2 h. The extracts were centrifuged at 11,000 rpm for 20 min. The supernatants were collected and then dried with an N_2_ stream at ambient conditions. The dried residues were reconstituted in 1 ml methanol for HPLC-MS/MS analysis.

### Relative quantification of selected cardenolides by HPLC-MS/MS

HPLC-MS/MS was performed using a Q Exactive HF-X Orbitrap mass spectrometer (Thermo Fisher Scientific, Bremen, Germany) coupled to a Dionex UltiMate 3000 HPLC instrument (Thermo Fisher Scientific, MA, USA). Analytes were separated on a Kinetex® C18 reversed-phase column (2.6 μm, 100 × 2.1 mm, Phenomenex, Torrance, USA). The injection volume was 15 μl, and the column compartment was set to 30 °C. Mobile phase A was water (0.1% FA) and mobile phase B was acetonitrile (0.1% FA) at a flow rate of 0.5 ml/min applying the following gradient: 0−2 min, 10% B; 2−20 min, 20−70% B; 20−25 min, 70−95% B; 25−30 min, 95% B; 30−35 min, 95−10% B. The mass spectrometer was operated in positive-ion mode in a mass-to-charge (*m*/*z*) range of 250 to 1000 at a mass resolution of 240,000 at *m*/*z* 200. Using HESI-source, following parameters were applied: spray voltage (+), 3.5 kV; capillary temperature, 300 °C; sheath gas flow rate, 35 psi; aux gas flowrate, 12 psi; aux gas heater temperature, 150 °C. HCD method with a collision energy of 25 eV was used for fragmentation. In total, three biological replicates of each injured and intact leaf samples were analysed, and the averaged peak area intensity was compared to determine the mean differences in the cardenolide content.

### Data processing and image generation

Xcalibur (Thermo Fisher Scientific, MA, USA) was used to display mass spectra. Ion images of selected *m*/*z* values were generated using MIRION imaging software [24] with a mass bin width of *m*/*z* ± 5 ppm from the exact mass. MS images were normalised to the highest intensity measured for each ion separately. No further image processing steps such as smoothing or TIC normalisation were used. RGB MS images were obtained by selecting and overlaying three different *m*/*z* values for the red-green-blue channels. Metabolites were assigned and identified in a combination of exact mass measurements, MS/MS experiments, METASPACE annotations [25] and METLIN Metabolite Database search [26]. The mass accuracy of metabolites in the imaging experiments was calculated in root mean square error (RMSE) for each mass occurring in the entire measurement.

## Results and discussion

### Visualising cardenolides in the intact leaf tissue of *A. curassavica*

**Fig. 1:**
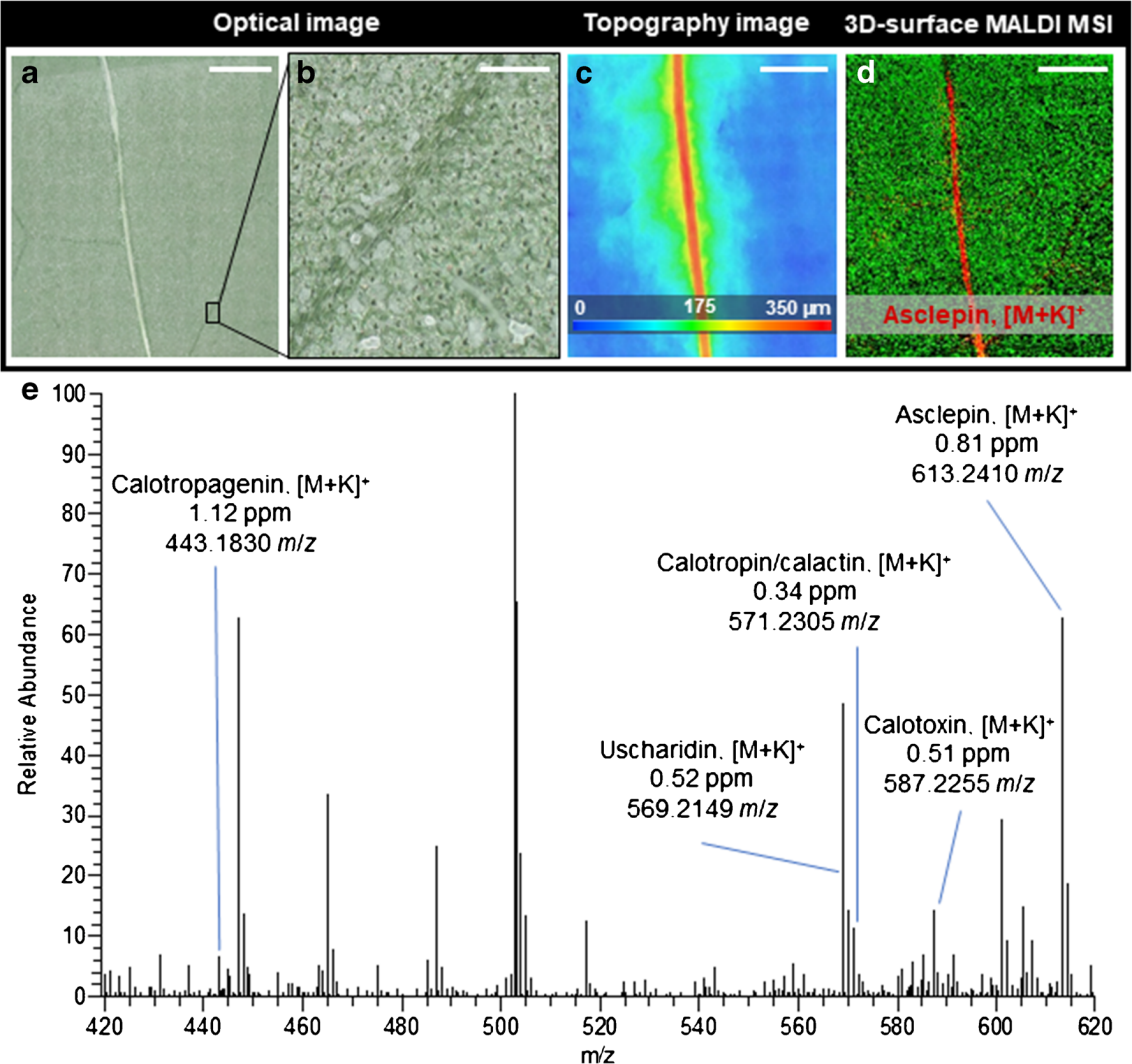
3D-surface MALDI MS imaging of an intact *A. curassavica* leaf. **a** Optical microscope image of the leaf surface after measurement. **b** Magnification (× 8) showing laser ablation spots on the leaf surface. **c** Topography image of an intact *A. curassavica* leaf surface showing height variations of up to 350 μm (leaf height varies from ‘cold’ (blue) to ‘hot’ (red). **d** Red-green overlay MS image of asclepin (*m*/*z* 613.2410, [M + K]^+^, red) and disaccharide at (*m*/*z* 381.0793, [M + H]^+^, green). **e** Single pixel mass spectrum for mass range *m*/*z* 420–620, obtained from the vein area of the leaf. Six different cardenolides are labelled with measured mass-to-charge-number ratio, compound name and mass deviation. MS images were generated with 178 × 178 pixels, 45 μm pixel size, *m*/*z* bin width: Δ(*m*/*z*)*/m/z* = ± 5 ppm. The scale bars are **a**, **c**, **d** 1 mm and **b** 150 μm

**Fig. 2:**
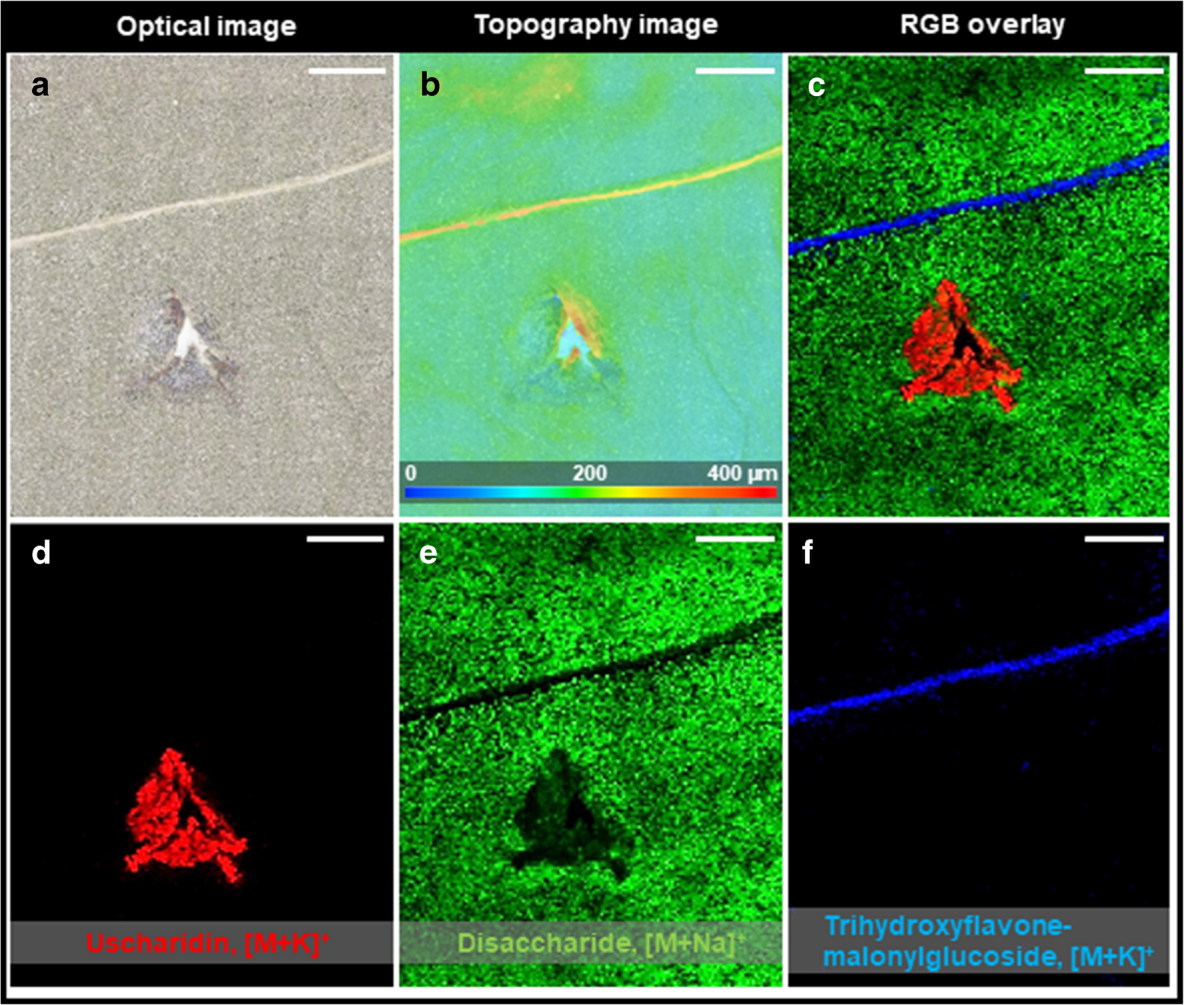
3D-surface MALDI MS imaging of an injured leaf of *A. curassavica* after 1 h*.* **a** Optical microscope image of the leaf surface after measurement. **b** Topography image of the leaf surface. The injured parts are showing height differences up to 400 μm. **c** RGB overlay of ion images showing the spatial distribution of uscharidin (*m*/*z* 553.2411, [M + K]^+^, red), disaccharide (*m*/*z* 365.1056, [M + Na]^+^, green), and trihydroxyflavone-malonylglycoside (*m*/*z* 557.0692, [M + K]^+^, blue). **d** Single ion image of uscharidin (*m*/*z* 553.2411, [M + K]^+^). **e** Single ion image of disaccharide (*m*/*z* 365.1056, [M + Na]^+^). **f** Single ion image of trihydroxyflavone-malonylglycoside (*m*/*z* 557.0692, [M + K]^+^). MS images were generated with 143 × 171 pixels; 35 μm pixel size; *m*/*z* bin width: Δ(*m*/*z*)*/m/z* = ± 5 ppm. All scale bars are 1 mm

Before 3D-surface MALDI MS imaging of wounded *A. curassavica* leaf samples, we analysed the chemical composition and spatial distribution of cardenolides on intact leaf samples using 3D-surface MALDI MS imaging. Figure 1a shows the optical image of an intact *A. curassavica* leaf surface after measurement. A detailed magnification of uniform laser ablation spots on the matrix-covered leaf surface is depicted in Fig. 1b, demonstrating that several cell layers were penetrated. Notably, no “volcano-like” matrix ejection (possibly causing analyte delocalisation) from the laser ablation spots was observed. *A. curassavica* leaf surfaces are uneven, with height variations up to 300 μm at secondary leaf veins (Fig. 1c) and up to 350 μm at primary leaf veins and wounded plant tissue (Fig. 2b). To investigate these irregular leaf surfaces and characteristic features via MALDI MS imaging, an autofocusing MALDI imaging source is indispensable. Besides chemical information, this method also provides topographic information allowing researchers to create topography images (see ESM Fig. S2 for comparison of the optical topography image generated from the MALDI ion source to the image from a digital 3D optical microscope). The chemical and topographic information can also be combined into a 3D-surface RGB MS image (ESM Fig. S3). A mass spectrum acquired from a single 45-μm pixel at the midvein is shown in Fig. 1e, highlighting six different cardenolides in the mass range of *m*/*z* 420 to 620 (see Table 1 for all detected cardenolides). To reduce the measurement time, the pixel size was not set to the smallest possible value for a given image size. Nevertheless, the selected pixel size of 20 to 45 μm was found to be appropriate for the biological question, regarding the spatial distribution of defence metabolites in the *A. curassavica* leaf. The optical image of the leaf surface (Fig. 1a) can be directly correlated to the corresponding ion image (Fig. 1d). The red colour channel in the ion image represents the distribution of the cardenolide asclepin (*m*/*z* 613.2410, [M + K]^+^). The green channel represents an unidentified disaccharide (*m*/*z* 381.0793, [M + K]^+^). The metabolites were assigned based on accurate mass measurements. For example, asclepin (*m*/*z* 613.2410, [M + K]^+^) was detected with a mass error of 0.81 ppm, and the root mean square error (RMSE), calculated from 31,684 spectra over the full image, was 1.29 ppm (see ESM Fig. S4 for RMSE plot for each cardenolide). Furthermore, the mass accuracy was better than 2 ppm for all compounds assigned in this study. Among the MALDI matrixes, 2,5-dihydroxybenzoic acid (2,5-DHB) gave the best results for cardiac glycosides and other secondary plant metabolites in comparison to different matrices such as 2-mercaptobenzothiazole (2-MBT) in positive-/negative-ion mode and 1,8-bis(dimethylamino)naphthalene (DMAN, “proton-sponge”) in negative-ion mode. In the intact leaf samples, it was possible to detect and assign seven different cardenolides such as calotropin or calactin (isomers) or uscharin that is known to commonly occur in *A. curassavica* [23]. All of these toxic metabolites showed an identical spatial distribution along the leaf vein, where non-articulated laticifer cells containing highly pressurized stores of latex are located (see Fig. 1d and ESM Fig. S5). Therefore, the MSI results confirm that cardenolides are primarily present in the latex. For instance, Züst et al. [27] reported 100-fold higher cardenolide concentrations in latex compared to leaves, suggesting that cardenolides accumulate in the latex and that concentrations are comparatively low in the leaf tissue. The latex is transported to the point of damage, providing a strong defence when and where it is needed the most.

**Table 1:** Selected cardiac glycosides from Zhang et al. [23] also assigned in wounded *A. curassavica* leaf by 3D-surface MALDI MS imaging. Metabolites were assigned, based on high mass accuracy of full scan MS data. In general, cardenolides were readily and mainly detected as [M + K]^+^ adduct ions in positive-ion mode

Compound	Chemical formula	Adduct	Exact mass (u)	RMSE (ppm)
Calotropagenin	C_23_H_32_O_6_	[M + K]^+^	443.1830	0.86
Uscharidin	C_29_H_38_O_9_	[M + K]^+^	569.2147	1.61
Calotropin/calactin	C_29_H_40_O_9_	[M + K]^+^	571.2304	1.52
Calotoxin	C_29_H_40_O_10_	[M + K]^+^	587.2253	1.27
Calactinic acid	C_29_H_40_O_11_	[M + K]^+^	603.2202	1.21
Asclepin	C_31_H_42_O_10_	[M + K]^+^	613.2410	1.29
Uscharin	C_31_H_41_NO_8_S	[M + K]^+^	626.2184	1.15
16α-Acetoxycalotropin	C_31_H_42_O_11_	[M + K]^+^	629.2359	1.23

### Visualising cardenolides in the wounded leaf of *A. curassavica*

The distribution of cardenolides after wounding (*t* = 1 h between wounding and harvesting) of an *A. curassavica* leaf was analysed using 3D-surface MALDI MS imaging. The optical microscope image (Fig. 2a) and the topography image (Fig. 2b) of an injured leaf surface after the measurement can be directly correlated to the corresponding ion images (Fig. 2c–f). The injured plant tissue was pointing upwards without any visible exudation of latex at the edges and showed height variations up to 600 μm. Figure 2c shows an overlay image, illustrating the spatial distribution of the cardenolide uscharidin (*m*/*z* 553.2411, [M + Na]^+^, red) (Fig. 2d), disaccharide (*m*/*z* 365.1056, [M + Na]^+^, green) (Fig. 2e) and trihydroxyflavone-malonylglycoside (*m*/*z* 557.0692, [M + K]^+^, blue) (Fig. 2f). In total, nine different cardenolides were detected and assigned, based on accurate mass measurements (Table 1 and ESM Fig. S6). For instance, uscharidin (*m*/*z* 553.2411, [M + K]^+^) (Fig. 2c) was assigned, based on accurate mass with a root mean square error (RMSE), calculated from 24,453 spectra over the full image, of 0.31 ppm (see ESM Fig. S3 for RMSE plot for each cardenolide). As shown in Fig. 1c for uscharidin ([M + K]^+^, *m*/*z* 553.2411) and ESM Fig. S6 for all remaining cardenolides, the toxic glycosides were exclusively detected in plant tissue very close (0.5 to 1 mm) to the injured area of the leaf, which is evident from overlaying microscopic and RGB MS images (ESM Fig. S7). Furthermore, this specific spatial distribution of cardenolides was reproducible for an additional biological replicate (i.e. leaves from a different plant) under identical experimental conditions, as shown in ESM Fig. S8. As in the measurement shown, all detected cardenolides display the same spatial distribution, i.e. very close to the injured area of the leaf. To confirm the MSI results, the cardenolide content for intact and injured leaf discs (*n* = 3 for each) were analysed using HPLC-ESI-HRMS. After 120 min of wounding, the concentration of five different cardenolides (ESM Fig. S9 for MS/MS spectra; ESM Fig. S10 for chromatograms) increased to 2.6- to 9-fold in the region of damaged leaf tissue (see ESM Fig. S9). Thus, both the MSI and the LC-MS data are supporting the hypothesis that wounding increases the latex flow rate towards the point of damage, and subsequently toxic compounds (e.g. cardenolides) accumulate in the wounded tissue. Despite both methods provide strong evidence, potential matrix or suppression effects improving the ionisation of cardenolides in wounded plant tissue cannot be excluded. Alternatively, or in addition, an increase of synthesis of cardenolides upon induction could contribute to the observed differences [28]. As we observed high cardenolide concentrations at the site of wounding already after 10 min, however, it seems unlikely that increased cardenolide synthesis explains the observed pattern. Therefore, our results support previous research suggesting that latex is an allocable defence [29, 30] and for the first time, we visualised rapid translocation of cardenolides to the site of damage.

### Analysing the rate of cardenolide accumulation overtime via 3D-surface MALDI MS imaging

The clotting activity of latex to prevent the outflow of latex is essential to maintain the plant’s chemical defence. Thus, pressure in the laticifers is upheld by sealing the wounds that theoretically halt the transport of toxic cardenolides at the point of damage. The cardenolide concentration and distribution were different depending on time after injury. To investigate the rate of cardenolide accumulation over time and space, we employed 3D-surface MALDI MS imaging to analyse an *A. curassavica* leaf sample that was wounded twice (spatially separate) at a time interval of 290 min.

**Fig. 3:**
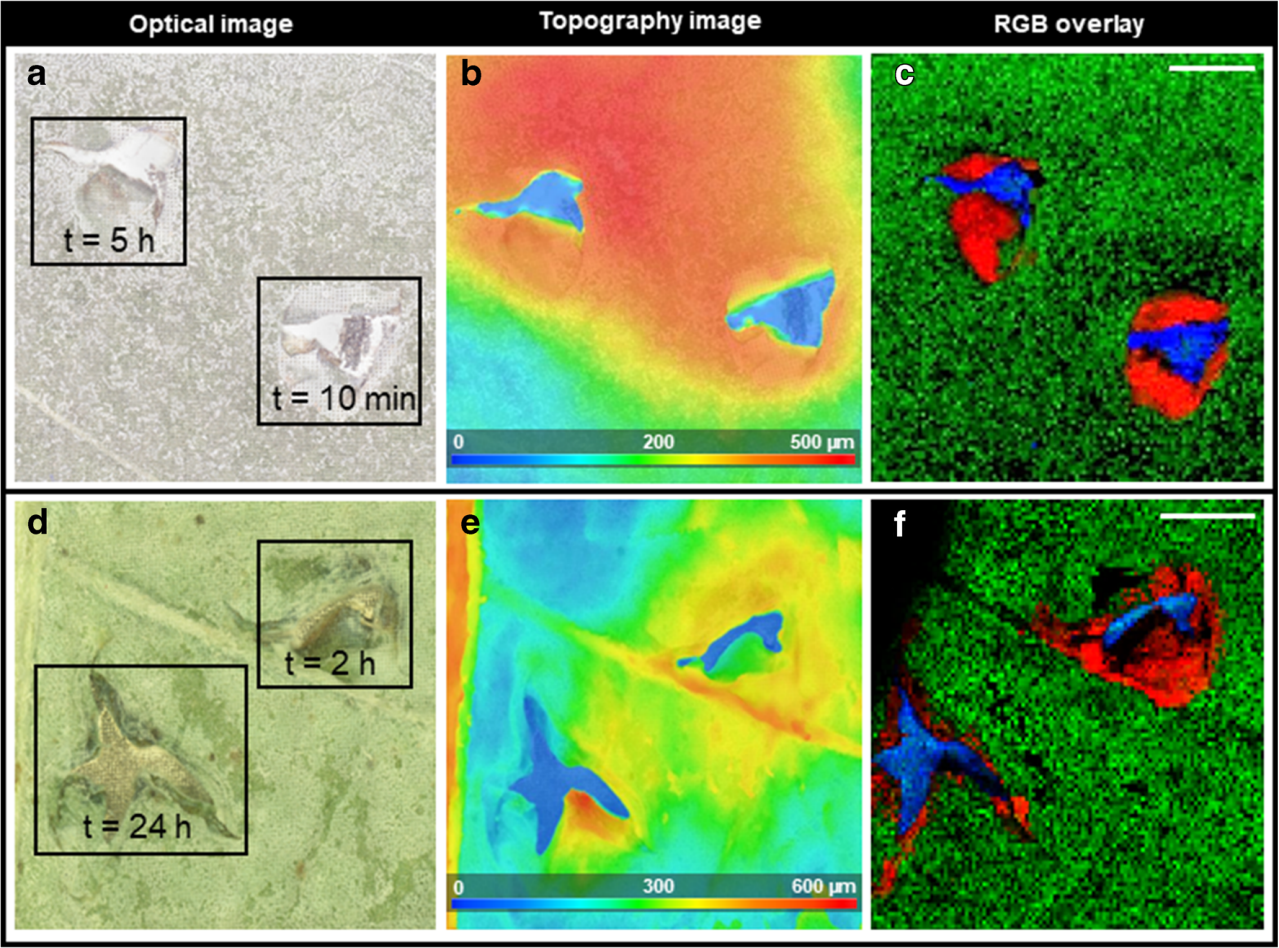
3D-surface MALDI MS imaging of *A. curassavica* leaf. Both samples were injured twice at a different time interval before harvesting. **a**, **d** Optical microscope image of the leaf surface after measurement. The injured areas are marked and associated time interval is indicated. **b**, **e** Topography image of the leaf surface showing height differences up to 500 μm and 600 μm, respectively. **c**, **f** RGB overlay of ion images showing the spatial distribution of calotoxin (*m*/*z* 587.2250, [M + K]^+^, red), dihydroxyflavone (*m*/*z* 255.0652, [M + H]^+^, green) and *m*/*z* 255.2110 (blue). MS images were generated with **c** 111 × 111 pixels, 45 μm pixel size, **f** 112 × 103 pixels; 35 μm pixel size; **c**, **f** *m*/*z* bin width: Δ(*m*/*z*)*/m/z* = ± 5 ppm. The scale bars are 1 mm

The optical microscopic image (after measurement) of an injured leaf sample (*t* = 5 h, *t* = 10 min) is depicted in Fig. 3a. The corresponding topography image of the leaf surface (Fig. 3b) demonstrates height variations up to 500 μm around the injured areas. Figure 3c is an overlay image of three selected ion images showing different structures of the injured leaf. Cardenolides like calotoxin (*m*/*z* 587.2250, [M + K]^+^, red) were exclusively located in both injured areas. Dihydroxyflavone (*m*/*z* 253.2639, [M + H]^+^, green) showed a uniform distribution on the entire leaf surface. A background signal (*m*/*z* 255.2110) from the MALDI metal target is shown in blue. Although the first injury (*t* = 5 h) was 290 min earlier than the second injury (*t* = 10 min), we did not observe substantial differences regarding the cardenolide content (see ESM Fig. S11 for all detected cardenolides). Thus, at the point of damage, latex exudation leads to rapid accumulation of defence compounds independently of previous damage on the same leaf (unless the main laticifers are not affected, see below). This makes sense in a way that a delayed defence mechanism would not have any impact against mechanical wounding or attacks from herbivorous insects because the time of feeding and consuming is relatively short. In order to test for possible changes regarding composition and amount of defence substances over an extended time span, the interval between the injuries was increased to 22 h. Figure 3d shows the optical microscopic image of a leaf sample (*t* = 24 h, *t* = 2 h, after wounding). We found that, after 24 h, the signal intensity was reduced compared to the site of fresh damage (measurement after 2 h), indicating that cardenolides are removed or degraded by the plant after a more extended period (previously demonstrated for *Calotropis procera* using LC-MS [31]). However, we did observe any degraded cardenolide products. The topographic image of the leaf surface is depicted in Fig. 3e showing height variations up to 600 μm along leaf veins and damaged leaf tissue. Hence, these characteristic features can only be analysed via an autofocusing MALDI ion source. Figure 3f shows 3D-surface MALDI MS imaging results for three selected ion signals that can be correlated to the optical image. The red colour channel represents the distribution of calotoxin (*m*/*z* 587.2250, [M + K]^+^, red). The green colour channel represents dihydroxyflavone (*m*/*z* 253.2639, [M + H]^+^, green), and *m*/*z* 255.2110 as a background signal of the MALDI target is shown in blue. As in previous experiments, calotoxin (*m*/*z* 587.2250, [M + K]^+^, red) shows a wide spatial distribution around injured leaf tissue after 2 h (see Fig. 3f). However, after 24 h, the typical spatial distribution around the entire injured area cannot be observed anymore. Instead, calotoxin (*m*/*z* 587.2250, [M + K]^+^, red) can only be detected on the inner edges of the damaged leaf tissue (see Fig. 3f). Thus, a decrease in cardenolide concentration can be visually observed after 24 h. This result was similar to all the detected cardenolides (see ESM Fig. S12) and demonstrates how the clotting activity of latex may avoid wasting defence compounds that require large amounts of resources for production. Hence, the latex flow rate towards the point of damage is reduced, and accumulated cardenolides are either delocalised or metabolised over time.

### Visualising the effect of a vein-cut regarding the spatial distribution of cardenolides via 3D-surface MALDI MS imaging

**Fig. 4:**
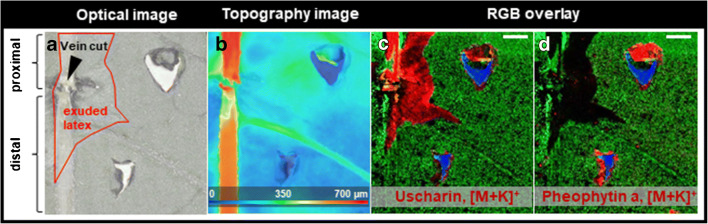
3D-surface MALDI MS imaging of *A. curassavica* leaf that was wounded proximal and distal after vein-cutting. **a** Optical microscopic image of the leaf surface before the measurement. Area of exuded latex on the leaf surface is marked. **b** Topographic image of the leaf surface showing height differences up to 700 μm at the primary leaf vein. **c**, **d** RGB overlay of ion images showing the spatial distribution of **c** uscharin (*m*/*z* 626.2189, [M + K]^+^, red), **d** pheophytin a (*m*/*z* 909.5291, [M + K]^+^, red), **c**, **d** malonylgenistin (*m*/*z* 557.0692, [M + K]^+^, green) and a background signal of the MALDI target **c**, **d** at *m*/*z* 771.4869 (blue). MS images were generated with 157 × 171 pixels; 40 μm pixel size; *m*/*z* bin width: Δ(*m*/*z*)*/m/z* = ± 5 ppm. The scale bars are 1 mm

Many herbivorous insects have evolved strategies to avoid latex-based plant defence by inactivating latex transport via laticifers. For instance, a single vein-cut while feeding can inactivate downstream latex flow [32–35]. Thus, defence substances can no longer be mobilised and accumulated at leaf parts; a herbivore has selected for feeding. After encountering minimal latex exudation during feeding, some insects even return to their initial cut to start vein-cutting again before resuming feeding [36]. Possible effects of fresh and clotted latex causing this counter-strategy have been investigated in previous studies. However, the chemical trigger for vein-cutting has not been identified yet [36]. In order to analyse and visualise the effects of vein-cutting regarding the spatial distribution of defence substances via 3D-surface MALDI MSI, *A. curassavica* leaves were mechanically wounded proximally to and distally to a vein-cut. Figure 4a shows the optical microscope image of the leaf surface. Exuded latex can be visually observed only around the severed leaf vein, but not at both the injuries. The topography image is depicted in Fig. 4b and displays height variations up to 700 μm at the primary vein. Figure 4c illustrates the spatial distribution of uscharin (*m*/*z* 626.2189, [M + K]^+^, red), malonylgenistin (*m/z* 557.0692, [M + K]^+^, green) and a background signal of the MALDI target at *m/z* 771.4869 (blue). As expected, cardenolides (such as uscharin) were predominantly detected around the damaged leaf vein, where latex exuded. Differences regarding the quantity of cardenolides at both injuries can be observed. Proximal to the vein-cut (i.e. before the vein-cut towards the plant), more extensive accumulation of uscharin (*m*/*z* 626.2189, [M + K]^+^) can be observed, in comparison to the distal leaf parts. This observation was made for all detected cardenolides. In order to display a chemical compound that is independent of latex exudation, Fig. 4d shows the spatial distribution of pheophytin a (*m*/*z* 909.5291, [M + K]^+^, red). The green and blue ion channels are the same as in Fig. 4c. Pheophytin a does not have any known defensive activity against herbivores, instead serves as an electron carrier intermediate in the electron transfer pathway of photosystem 2 (PS 2) and could not be detected in exuded latex on the leaf surface (i.e. latex does not contain this chemical compound). Thus, the spatial distribution of pheophytin a (*m*/*z* 909.5291, [M + K]^+^) is not affected by the vein-cut and shows high similarity regarding concentration for the proximal and distal injury, respectively. This experiment was repeated with a biological replicate, showing the same results for all compounds assigned previously (see ESM Fig. S13). In total, 3D-surface MALDI MS imaging demonstrated how vein-cutting affects the spatial distribution of defensive substances like uscharin (*m*/*z* 626.2189, [M + K]^+^), showcasing why the majority of specialised feeders for latex-containing plants like *A. curassavica* apply vein-cutting as a counter-strategy against possible intoxication while feeding on leaf parts.

## Conclusions

The AP-SMALDI5 AF imaging source coupled to the Q Exactive HF Orbitrap mass spectrometer provides high accuracy in mass and space. It allows investigating irregular 3D surfaces with a lateral resolution of ≤ 10 μm. We have employed this set-up for the identification and localisation of defence metabolites (here, cardenolides) and many other secondary metabolites in native *A. curassavica* leaf samples (no sectioning). We aimed to investigate a biological system providing an example of what is now possible with MS imaging of plant samples at this stage. Our results demonstrate the relevance and field of potential applications in the context of plant science. The autofocusing operation mode made it possible to image detailed topographic features with specific height variations. High spectral resolution and mass accuracy were necessary to resolve the peaks and assign metabolites based on accurate mass.

We were able to localise nine different cardenolides at the injured area of *A. curassavica* leaf samples, showing that latex is part of the chemical defence mechanisms against tissue damage and potential attacks by herbivorous insects. Upon mechanical wounding, latex flow rate towards the point of injury is increased, resulting in the accumulation of defence substances in the affected area of the leaf. This rate of accumulation was further analysed in time and space by quenching the plant’s defence mechanism after different time intervals (separating the leaf from the rest of the plant). Furthermore, we mimicked the insect’s strategy to overcome this defence mechanism (vein-cutting) observing less amount of toxic metabolites at the injury distal to the vein-cut. Throughout all experiments, a high number of unidentified ion signals showed the same spatial distribution as cardenolides, indicating a wide variety of defence substances in *A. curassavica* (ESM Fig. S14). Thus, untargeted metabolomics in the context of MS imaging with HPLC-MS as a complementary method can be more widely considered as a valuable method to discover and investigate plant metabolites of potential relevance for plant interactions with herbivorous insects and pathogens. In this context, performing on-tissue MALDI MS/MS will be helpful for the identification of unknown metabolites. With this experimental set-up capable of 3D-surface MALDI MS imaging, topographic features with height variations up to 700 μm in damaged leaf veins can be visualised with high lateral resolutions. In this respect, high-resolution 3D-surface MALDI MSI will be helpful to obtain molecular information at the cellular level concerning topographic features in plant science.

## Supplementary information

ESM 1 (PDF 2741 kb)

## Data Availability

All MS image files are available from the METASPACE database (https://metaspace2020.eu/project/DD_Asclepias_3DMSI).
