# Supplementary material for: 3D-surface MALDI mass spectrometry imaging for visualising plant defensive cardiac glycosides in Asclepias curassavica
Source: Anal Bioanal Chem. 2021 Feb 5;413(8):2125–34. doi: 10.1007/s00216-021-03177-y (PMC7943518; doi:10.1007/s00216-021-03177-y)
Supplement: Supplementary file 1 — (PDF 2741 kb) [file 216_2021_3177_MOESM1_ESM.pdf]

Supplementary Information

3D-surface MALDI mass spectrometry imaging for visualising plant defensive cardiac glycosides in *Asclepias curassavica*

Domenic Dreisbach[1], Georg Petschenka[2], Bernhard Spengler[1], Dhaka R. Bhandari[1*]

[1]Institute for Inorganic and Analytical Chemistry, Justus Liebig University Giessen, Heinrich-Buff-Ring 17, 35392 Giessen, Germany

[2]Institute of Phytomedicine, University of Hohenheim, Otto-Sander-Straße 5, 70599 Stuttgart, Germany

***Corresponding Author**

Dhaka Ram Bhandari
Institute of Inorganic and Analytical Chemistry
Heinrich-Buff-Ring 17
D-35392 Giessen
E-mail address: Dhaka.R.Bhandari@anorg.Chemie.uni-giessen.de

Table of contents

Sample preparation of injured and intact *A.curassavica* leaf samples for HPLC-MS S1

Comparison of topography images of an *Asclepias curassavica* leaf surface S2

Combination of chemical and topographic information into a 3D RGB ion image S3

RMSE plots for all detected cardenolides S4

RG ion images for seven different cardenolides detected in intact *Asclepias curassavica* leaf via 3D-surface MALDI MS imaging S5

RGB ion images for nine different cardenolides detected in injured (t = 1h) *Asclepias curassavica* leaf via 3D-surface MALDI MS imaging S6

Overlay of optical image and RGB MS image S7

The optical image, RGB MS image and detailed magnification of injured areas for two additional biological replicates of injured *Asclepias curassavica* leaf S8

HPLC-MS/MS of intact and injured *Asclepias curassavica* leaf discs S9

Chromatograms of five selected cardenolides used for relative quantification S10

RGB ion images for nine different cardenolides detected in two times injured (t_1 = 10 min; t_2 = 5h) *Asclepias curassavica* leaf via 3D-surface MALDI MS imaging S11

RGB ion images for nine different cardenolides detected in two times injured (t_1 = 2h; t_2 = 24h) *Asclepias curassavica* leaf via 3D-surface MALDI MS imaging S12

The optical image, topography image and RGB MS images for biological replicate of vein-cut and injured *Asclepias curassavica* leaf S13

Untargeted analysis of metabolites in the affected area for wounded Asclepias curassavica leaves via 3D-surface MALDI MS imaging S14

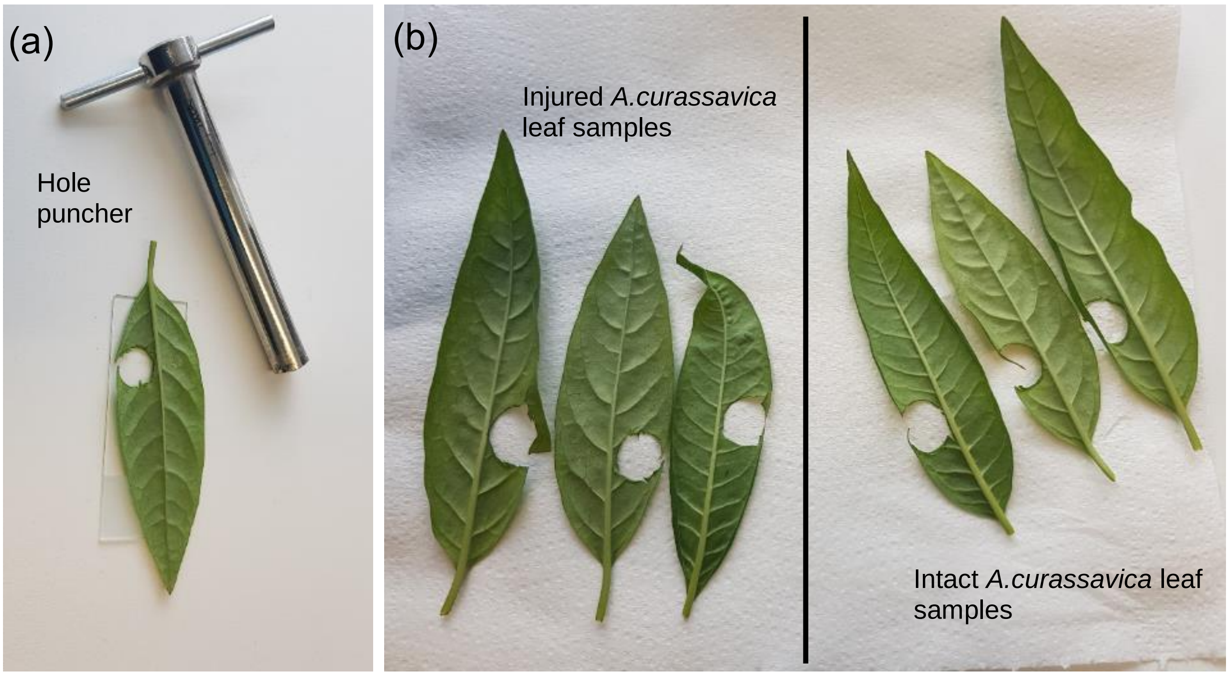

Figure S1. Sample preparation of injured and intact *A.curassavica* leaf samples for HPLC-MS. (a) Uniform leaf discs were obtained using a hole puncher. (b) Overview of three biological replicates for injured and intact *A.curassavica* leaf samples.

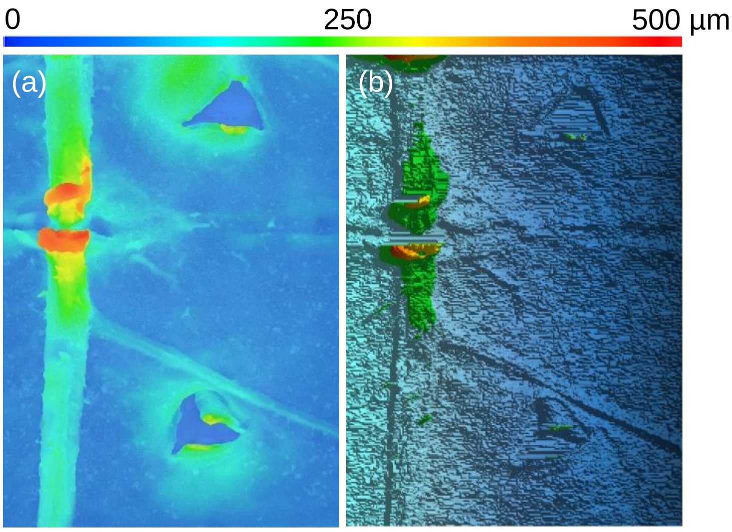

Figure S2. Comparison for topography images of an *Asclepias curassavica* leaf surface. (a) Image obtained via a 3D digital microscope. (b) Image generated via Excel based on the topographic data obtained.

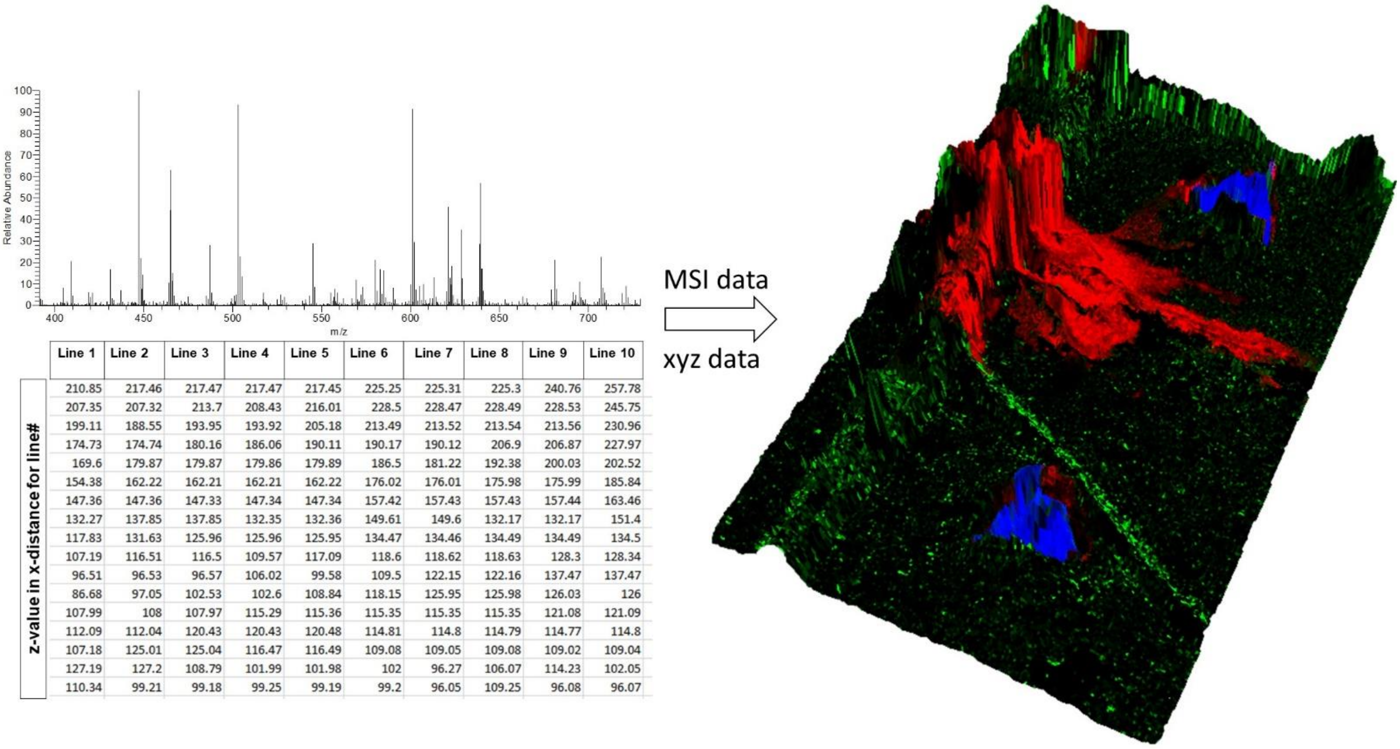

Line 1	Line 2	Line 3	Line 4	Line 5	Line 6	Line 7	Line 8	Line 9	Line 10
210.85	217.46	217.47	217.47	217.45	225.25	225.31	225.3	240.76	257.78
207.35	207.32	213.7	208.43	216.01	228.5	228.47	228.49	228.53	245.75
199.11	188.55	193.95	193.92	205.18	213.49	213.52	213.54	213.56	230.96
174.73	174.74	180.16	186.06	190.11	190.17	190.12	206.9	206.87	227.97
169.6	179.87	179.87	179.86	179.89	186.5	181.22	192.38	200.03	202.52
154.38	162.22	162.21	162.21	162.22	176.02	176.01	175.98	175.99	185.84
147.36	147.36	147.33	147.34	147.34	157.42	157.43	157.43	157.44	163.46
132.27	137.85	137.85	132.35	132.36	149.61	149.6	132.17	132.17	151.4
117.83	131.63	125.96	125.96	125.95	134.47	134.46	134.49	134.49	134.5
107.19	116.51	116.5	109.57	117.09	118.6	118.62	118.63	128.3	128.34
96.51	96.53	96.57	106.02	99.58	109.5	122.15	122.16	137.47	137.47
86.68	97.05	102.53	102.6	108.84	118.15	125.95	125.98	126.03	126
107.99	108	107.97	115.29	115.36	115.35	115.35	115.35	121.08	121.09
112.09	112.04	120.43	120.43	120.48	114.81	114.8	114.79	114.77	114.8
107.18	125.01	125.04	116.47	116.49	109.08	109.05	109.08	109.02	109.04
127.19	127.2	108.79	101.99	101.98	102	96.27	106.07	114.23	102.05
110.34	99.21	99.18	99.25	99.19	99.2	96.05	109.25	96.08	96.07

Figure S3. Combination of chemical and topographic data into a 3D RGB image generated via MIRION. Ion image shows the spatial distribution of $[asclepin+K]^+$ at *m*/*z* 613.2410 (red), $[malonylgenistin+K]^+$ at *m*/*z* 557.0692 (green) and a background signal of the MALDI target at *m*/*z* 771.4869 (blue).

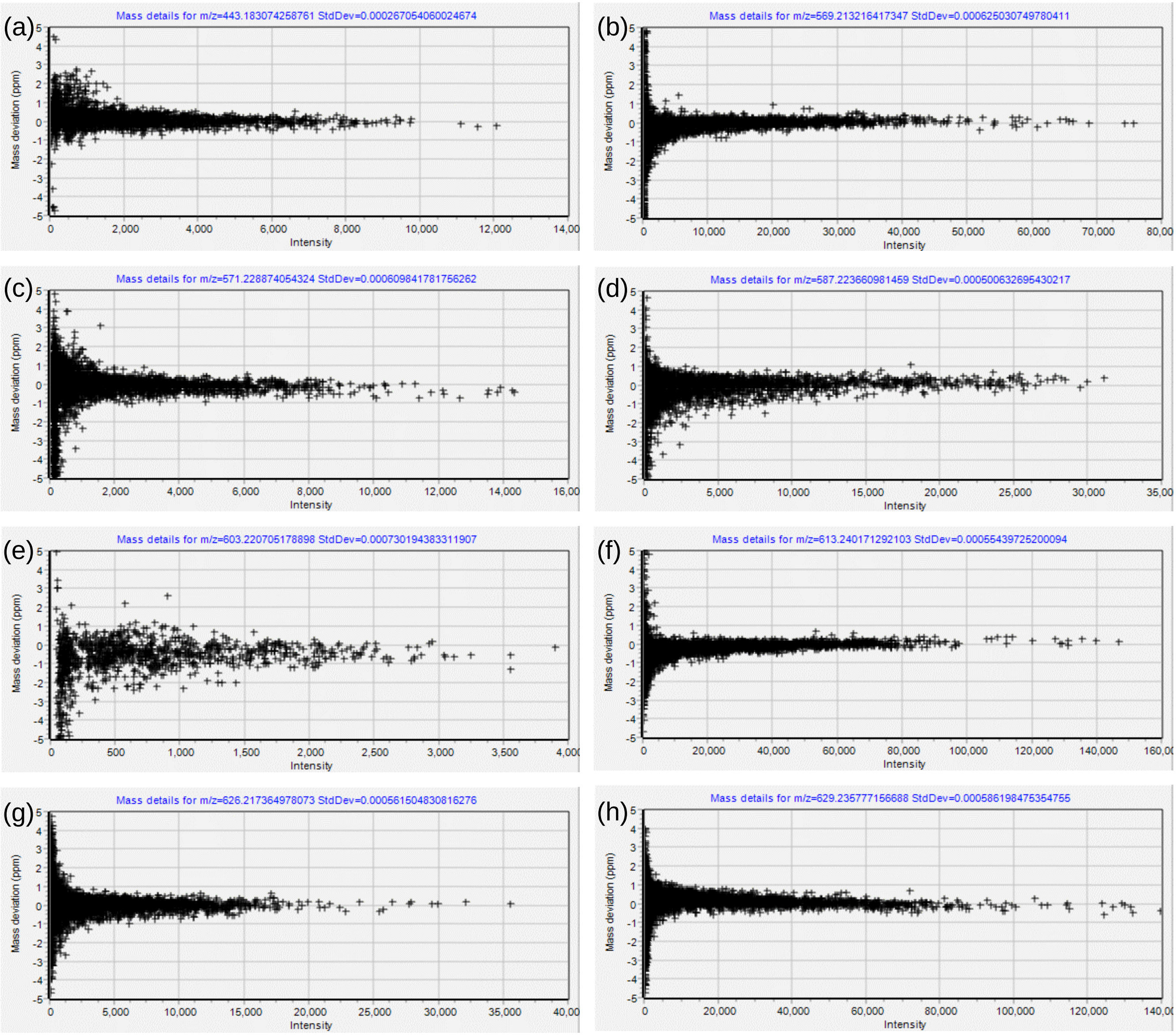

Figure S4. RMS plots for nine different cardenolides detected via 3D-surface MALDI MS imaging. (a) [calotropagenin+K]$^+$ at *m*/*z* 443.1830, (b) [uscharidin+K]$^+$ at *m*/*z* 569.2132, (c) [calotropin/calactin+K]$^+$ at *m*/*z* 571.2288, (d) [calotoxin+K]$^+$ at *m*/*z* 587.2236, (e) [calactinic acid+K]$^+$ at *m*/*z* 603.2207, (f) [asclepin+K]$^+$ at *m*/*z* 613.2401, (g) [uscharin+K]$^+$ at *m*/*z* 626.2173, (h) [acetoxycalotropin+K]$^+$ at *m*/*z* 629.2357.

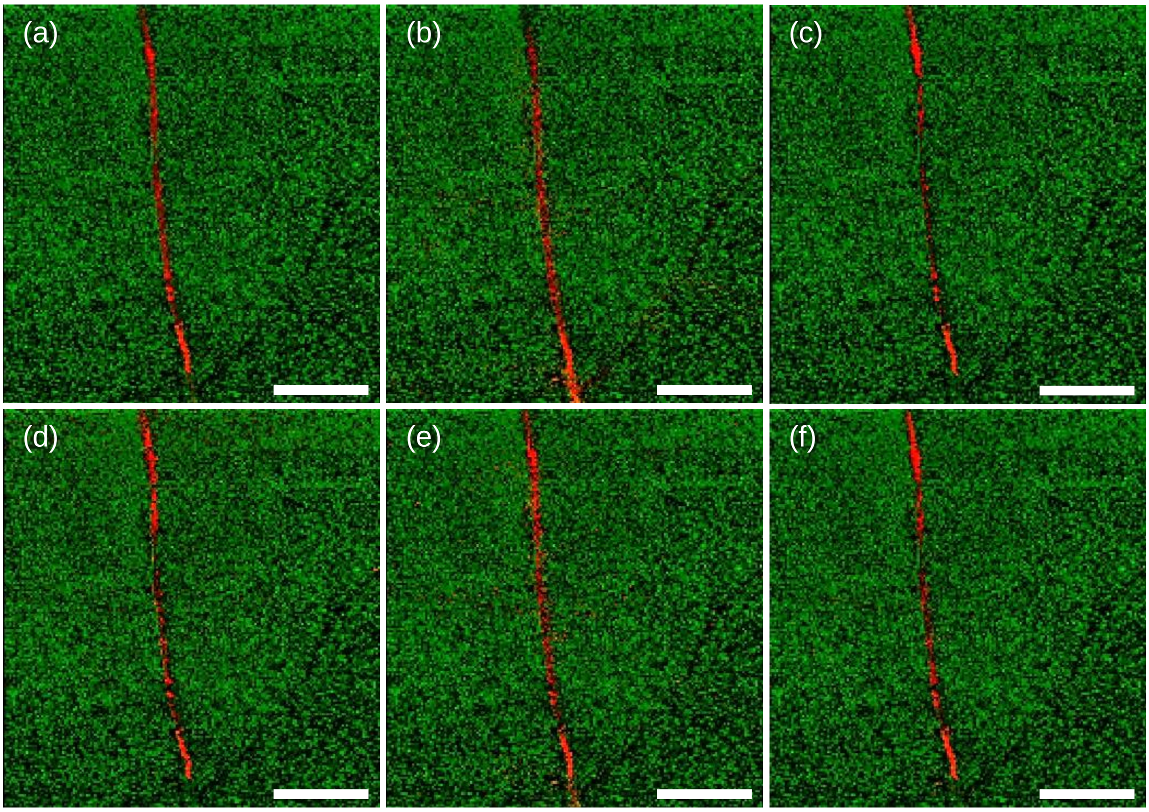

Figure S5. 3D-surface MALDI MS imaging of intact *Asclepias curassavica* leaf. RG ion images of (a) [calotropagenin+K]$^+$ at *m*/*z* 443.1830, (b) [uscharidin+K]$^+$ at *m*/*z* 569.2132, (c) [calotropin/calactin+K]$^+$ at *m*/*z* 571.2288, (d) [calotoxin+K]$^+$ at *m*/*z* 587.2236, (e) [calactinic acid+K]$^+$ at *m*/*z* 603.2207, (f) [uscharin+K]$^+$ at *m*/*z* 626.2173 in red and (a-f) [disaccharide+K]$^+$at *m*/*z* 381.0793 in green. MS images were generated with 178 x 178 pixels, 45 μm pixel size, *m*/*z* bin width: Δ(*m*/*z*) / *m*/*z* = ± 5 ppm. The scale bars are (a-f) 1 mm.

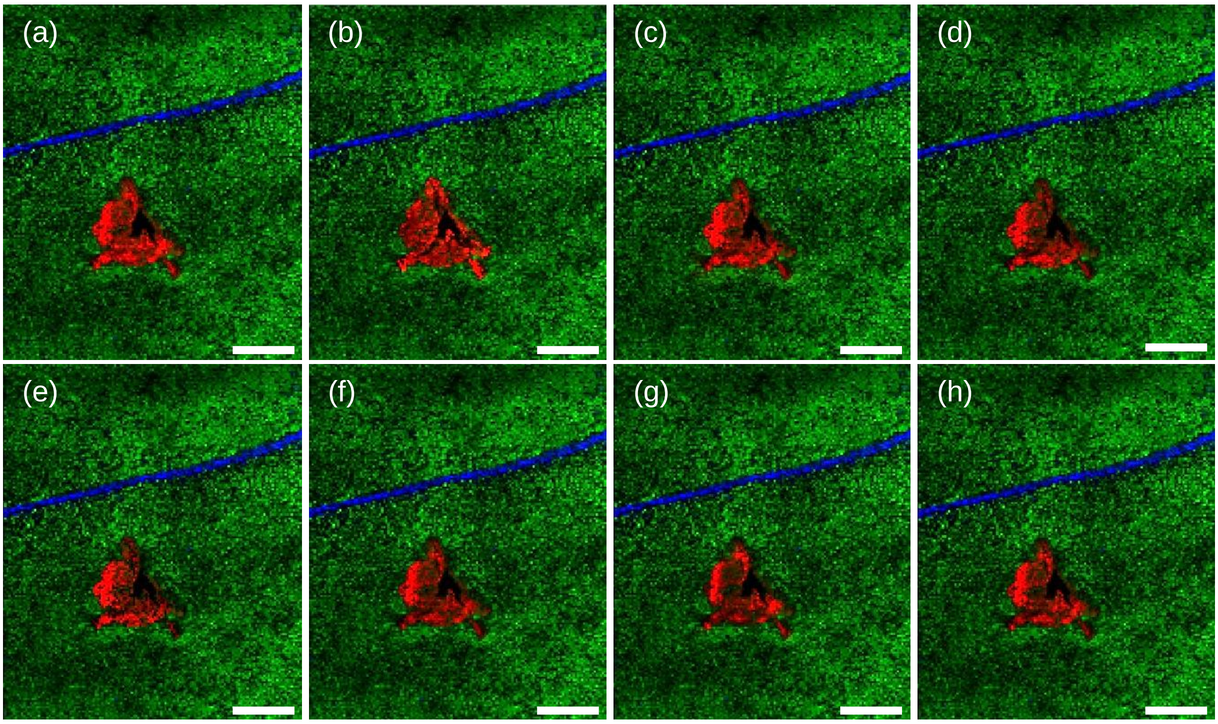

Figure S6. 3D-surface MALDI MS imaging of injured *Asclepias curassavica* leaf (t = 1h). RGB ion images of (a) [calotropagenin+K]$^+$ at *m*/*z* 443.1830, (b) [uscharidin+K]$^+$ at *m*/*z* 569.2132, (c) [calotropin/calactin+K]$^+$ at *m*/*z* 571.2288, (d) [calotoxin+K]$^+$ at *m*/*z* 587.2236, (e) [calactinic acid+K]$^+$ at *m*/*z* 603.2207, (f) [asclepin+K]$^+$ at *m*/*z* 613.2401, (g) [uscharin+K]$^+$ at *m*/*z* 626.2173, (h) [acetoxycalotropin+K]$^+$ at *m*/*z* 629.2357 in red, (a-h) [disaccharide+Na]$^+$ at *m*/*z* 365.1056 in green and (a-h) [trihydroxyflavone-malonylglycoside+K]$^+$ at *m*/*z* 557.0692 in blue. MS images were generated with 143 x 171 pixels; 35 µm pixel size; *m*/*z* bin width: Δ(*m*/*z*) / *m*/*z* = ± 5 ppm. All scale bars are 1 mm.

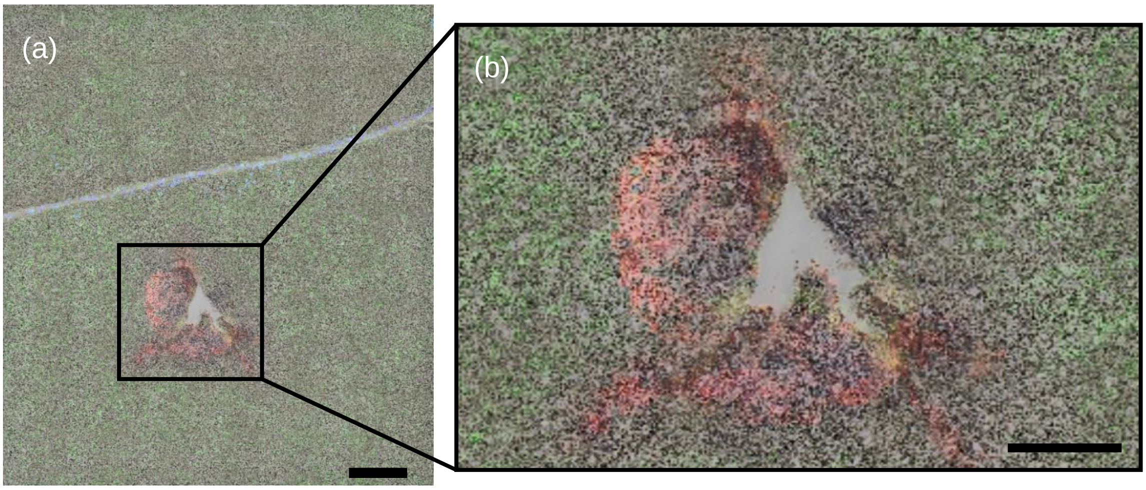

Figure S7. 3D-surface MALDI MS imaging of injured *Asclepias curassavica* leaf (t = 1h). (a) Overlay of optical image and RGB MS image from Figure 2c. (b) Detailed magnification of the injured area shows the spatial distribution of the cardenolide [uscharidin+Na]$^+$ in the damaged leaf parts. The scale bars are (a) 1 mm, (b) 300 µm.

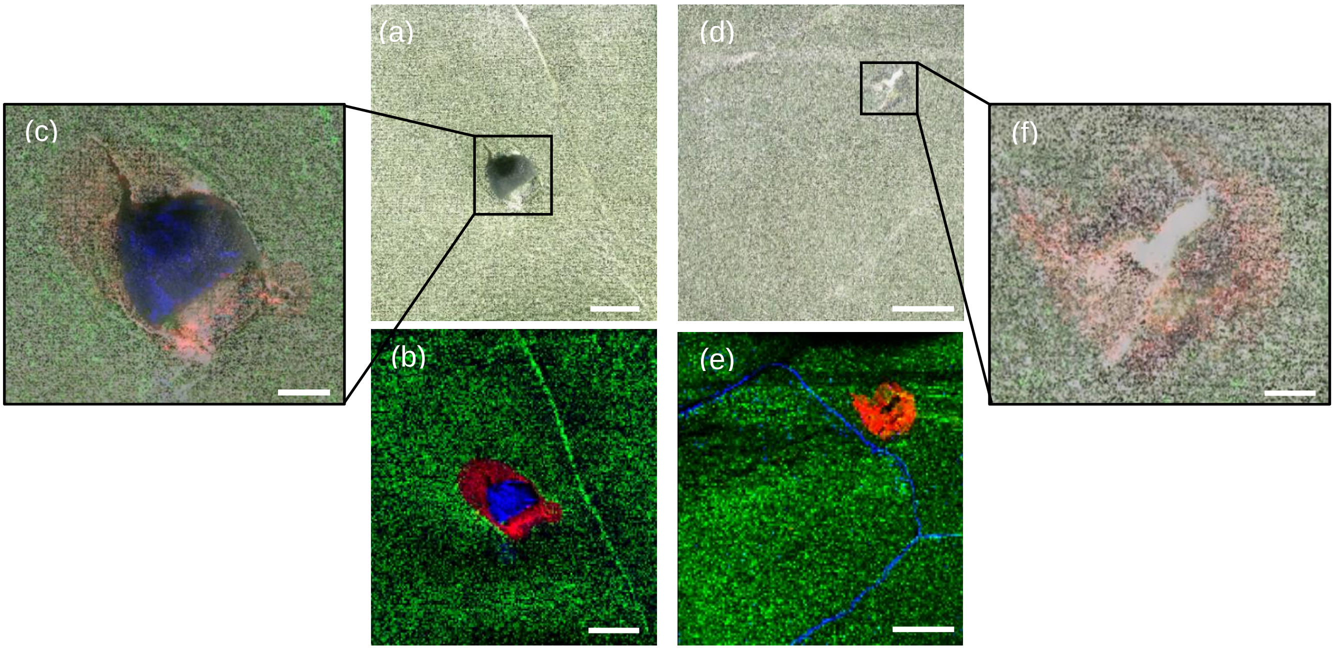

Figure S8. 3D-surface MALDI MS imaging of two additional biological replicates of injured *Asclepias curassavica* leaf (t = 1h). (a) Optical microscope image of the leaf surface after measurement. (b) RGB MS image of [asclepin+K]$^+$ at *m*/*z* 613.2401 (red), [dihydroxyspirilloxanthin+Na]$^+$ at *m*/*z* 651.4389 (green), [cichorrin+Na]$^+$ at *m*/*z* 363.0688 (blue). (c) Detailed magnification of the overlay of microscopic and RGB MS image. (d) Optical microscope image of the leaf surface after measurement. (e) RGB MS image of [uscharidin+K]$^+$ at *m*/*z* 569.2150 (red), [laricitrin-galactoside+Na]$^+$ at *m*/*z* 571.0960 (green) and background signal of the MALDI target at *m*/*z* 363.0688 (blue). (f) Detailed magnification of the overlay of microscopic and RGB MS image. MS images were generated with (b) 171 x 171 pixels (e) 150 x 150 pixels; 35 µm pixel size; *m*/*z* bin width: $\Delta(m/z) / m/z = \pm 5$ ppm. Scale bars are (a,b,d,e) 1 mm, (c) 350 µm, (f) 200 µm.

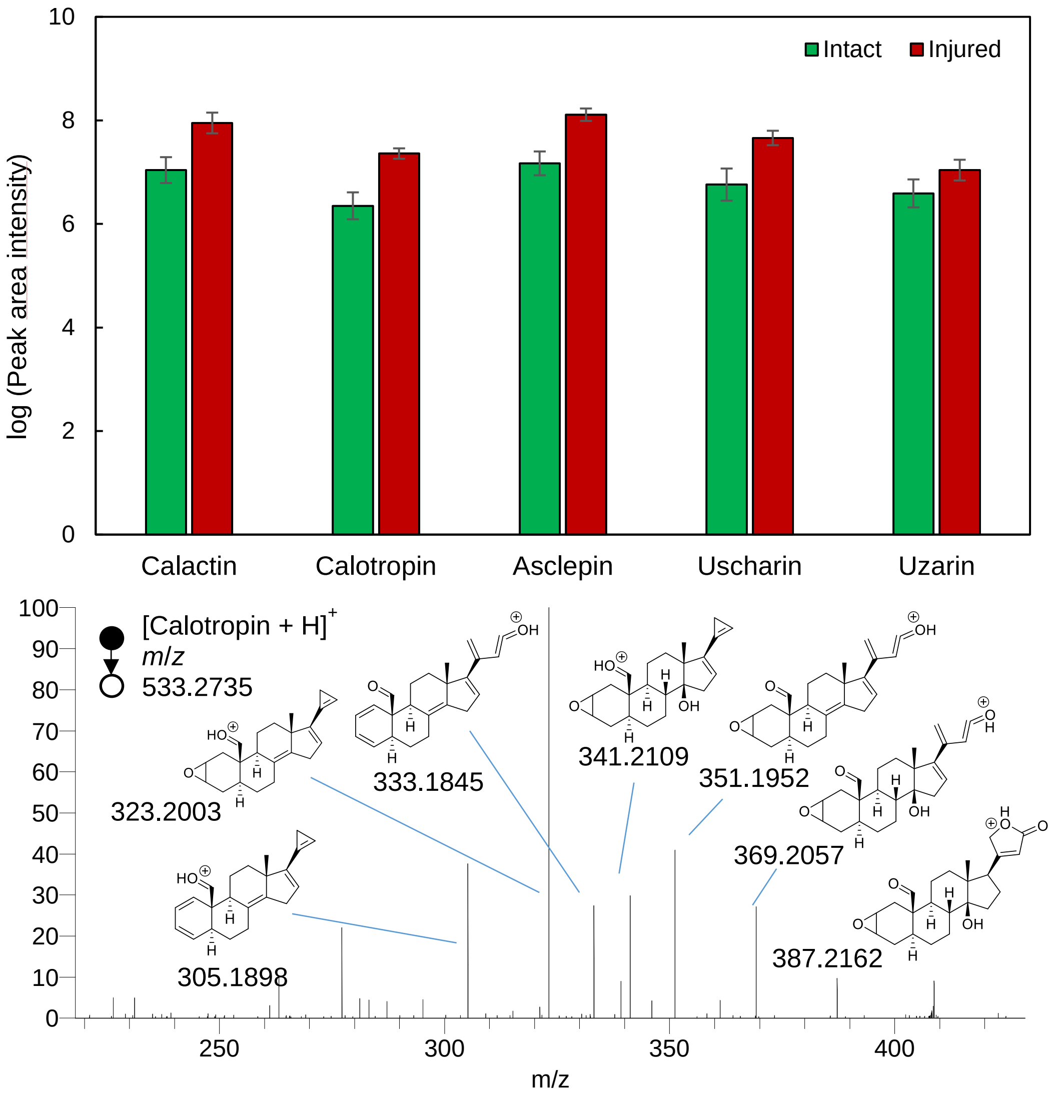

Figure S9. HPLC-ESI-MS of intact and injured *A. curassavica* leaf discs. (a) The logarithmic scale of the peak area intensity for five different cardenolides. For injured leaf discs, bulk cardenolide concentration increased to 2.6- to 9- fold. Each value is the mean of three biological replicates, and error bars indicate standard deviation. (b) All cardenolides were identified via MS/MS experiments and showed the same fragmentation pathway that consists of five characteristic fragments (shown for [Calotropin+H]$^{+}$).

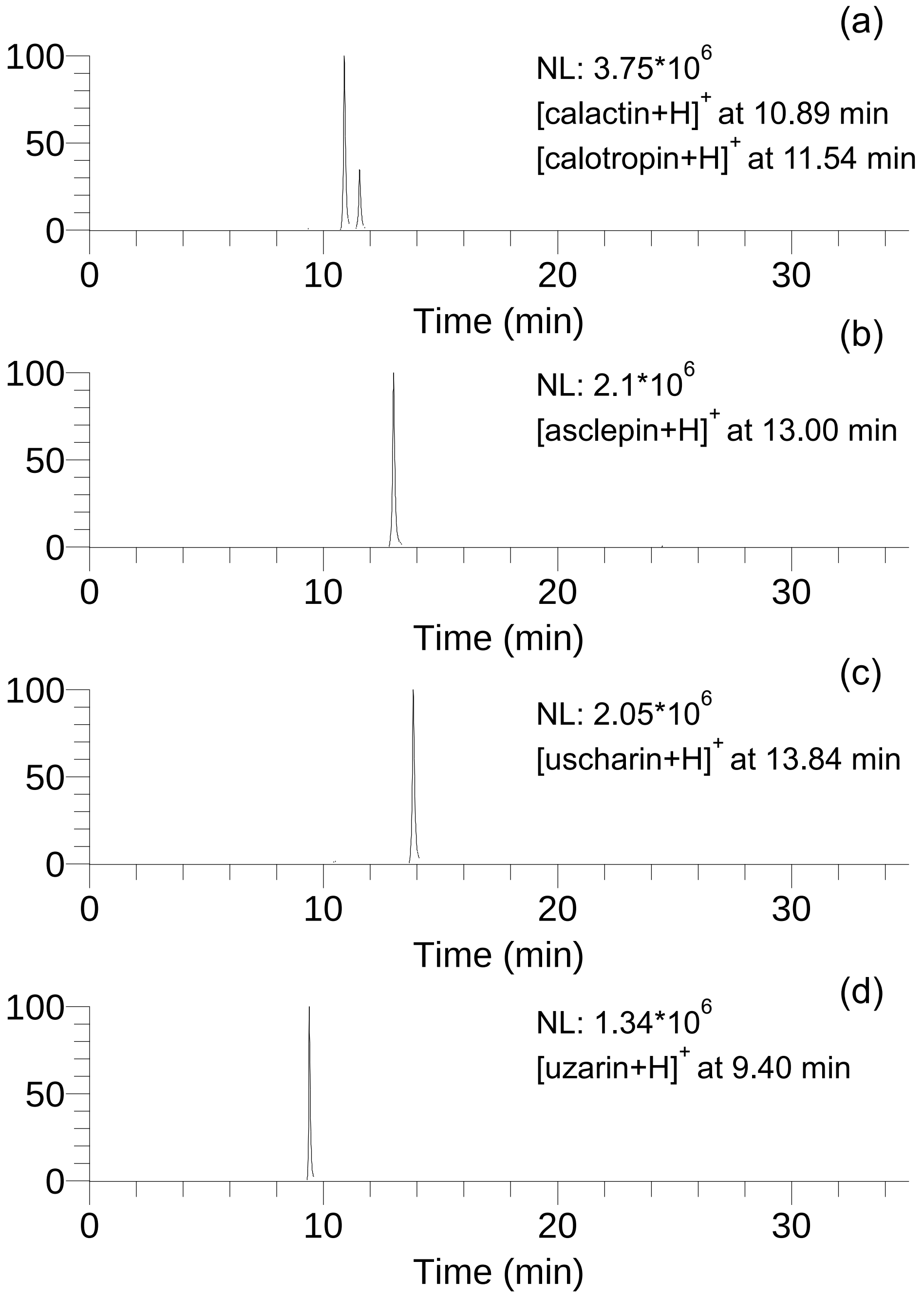

Figure S10. Chromatograms of five selected cardenolides used for relative quantification. (a) $[calactin/calotropin]^+$ at *m*/*z* 533.2751. (b) $[asclepin+H]^+$ at *m*/*z* 575.2866. (c) $[uscharin+H]^+$ at *m*/*z* 588.2651. (d) $[uzarin+H]^+$ at *m*/*z* 699.3583.

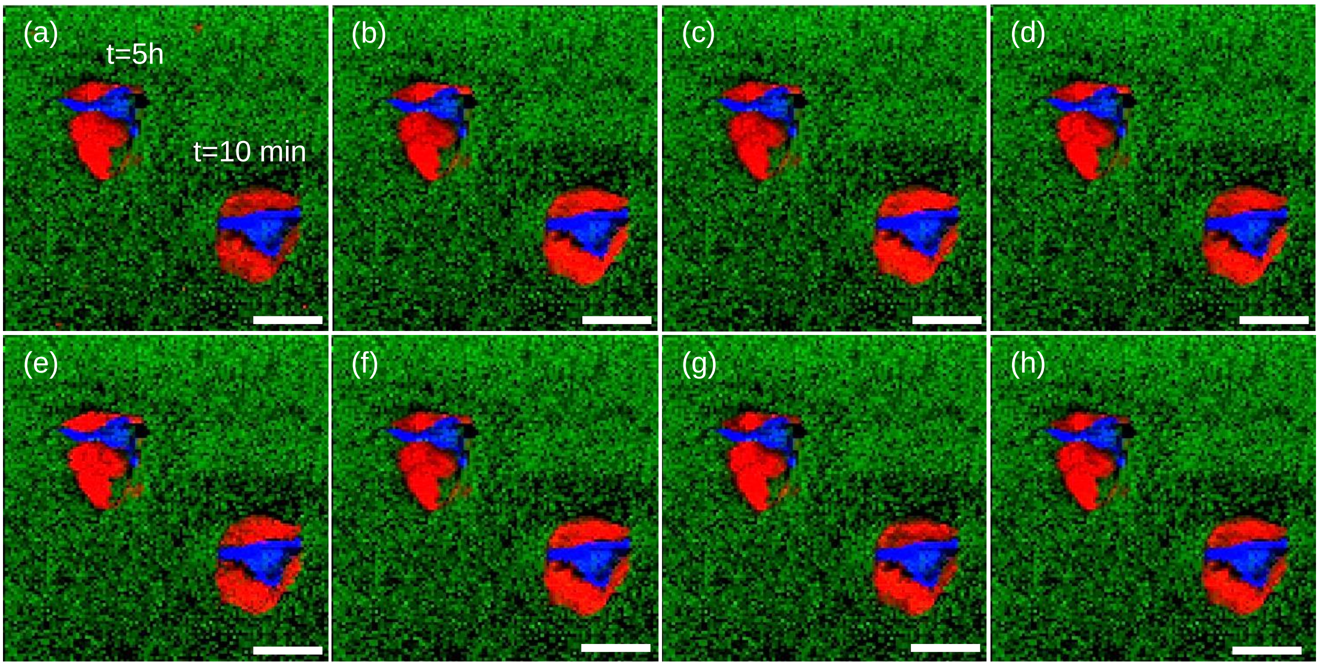

Figure S11. 3D-surface MALDI MS imaging of injured *Asclepias curassavica* leaf. The sample was wounded twice (spatially separate) but after different time points. RGB ion images of (a) $[calotropagenin+K]^+$ at *m*/*z* 443.1830, (b) $[uscharidin+K]^+$ at *m*/*z* 569.2132, (c) $[calotropin/calactin+K]^+$ at *m*/*z* 571.2288, (d) $[calotoxin+K]^+$ at *m*/*z* 587.2236, (e) $[calactinic\ acid+K]^+$ at *m*/*z* 603.2207, (f) $[asclepin+K]^+$ at *m*/*z* 613.2401, (g) $[uscharin+K]^+$ at *m*/*z* 626.2173, (h) $[acetoxycalotropin+K]^+$ at *m*/*z* 629.2357 in red, (a-h) $[swertisin+K]^+$ at *m*/*z* 485.0843 in green and (a-h) background signal of the MALDI target at *m*/*z* 255.2110 in blue. MS images were generated with 111 x 111 pixels; 45 µm pixel size; *m*/*z* bin width: $\Delta(m/z) / m/z = \pm 5$ ppm. All scale bars are 1 mm.

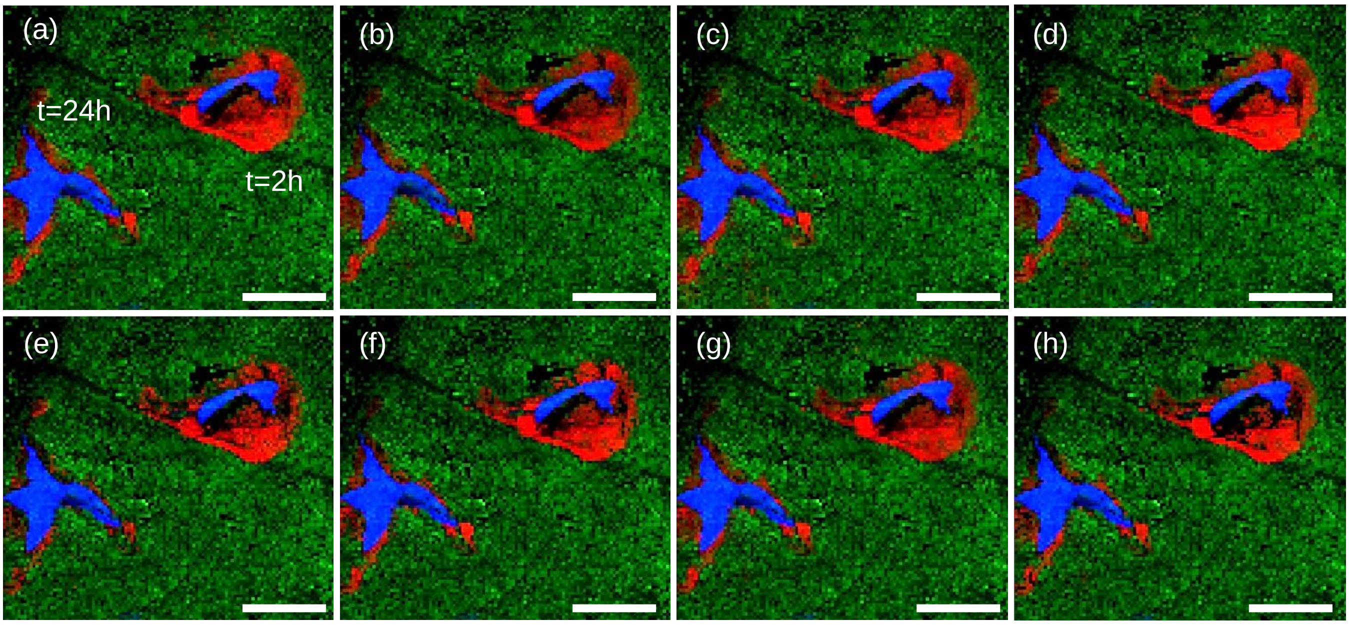

Figure S12. 3D-surface MALDI MS imaging of injured *Asclepias curassavica* leaf. The sample was wounded twice (spatially separate) but after different time points. RGB ion images of (a) $[calotropagenin+K]^+$ at *m*/*z* 443.1830, (b) $[uscharidin+K]^+$ at *m*/*z* 569.2132, (c) $[calotropin/calactin+K]^+$ at *m*/*z* 571.2288, (d) $[calotoxin+K]^+$ at *m*/*z* 587.2236, (e) $[calactinic\ acid+K]^+$ at *m*/*z* 603.2207, (f) $[asclepin+K]^+$ at *m*/*z* 613.2401, (g) $[uscharin+K]^+$ at *m*/*z* 626.2173, (h) $[acetoxycalotropin+K]^+$ at *m*/*z* 629.2357 in red, (a-h) $[dihydroxyflavone+H]^+$ at *m*/*z* 255.0652 in green and (a-h) background signal of the MALDI target at *m*/*z* 255.2110 in blue. MS images were generated with 112 x 103 pixels; 35 µm pixel size; *m*/*z* bin width: $\Delta(m/z) / m/z = \pm 5$ ppm. All scale bars are 1 mm.

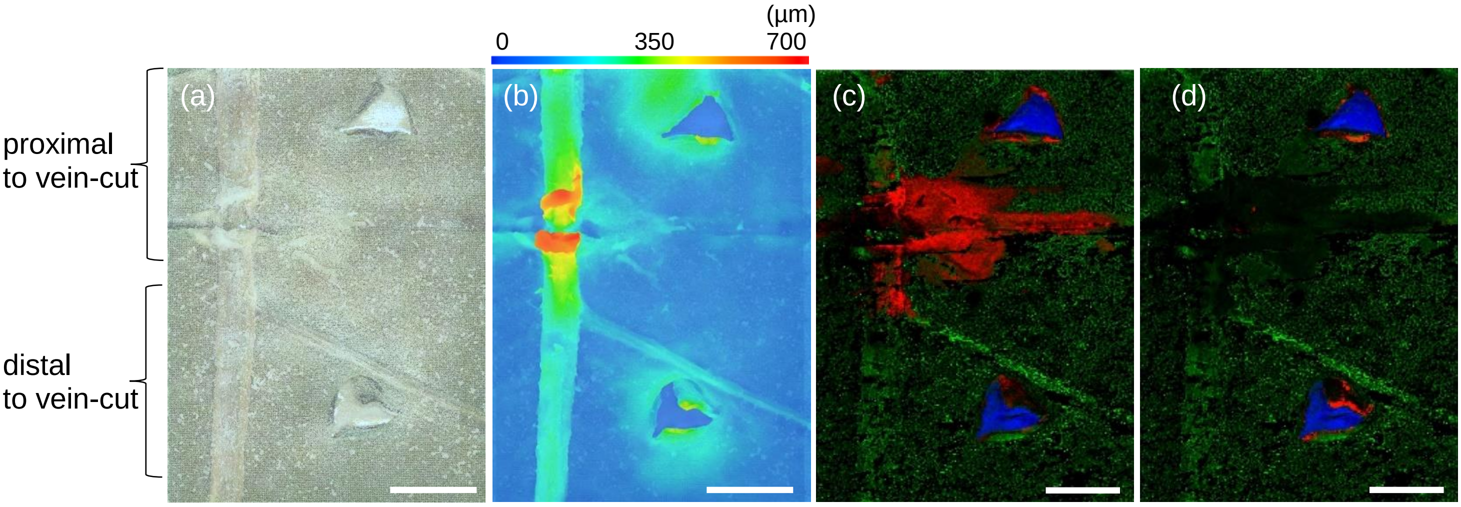

Figure S13. 3D-surface MALDI MS imaging of *Asclepias curassavica* leaf that was wounded proximal and distal to a vein-cut. (a) Optical microscopic image of the leaf surface after the measurement. (b) Topographic image of the leaf surface showing height differences up to 700 µm at the primary leaf vein. (c) Overlay of ion images showing the spatial distribution of $[\text{uscharin+K}]^+$ at *m*/*z* 626.2189 (red), $[\text{malonylgenistin+K}]^+$ at *m*/*z* 557.0692 (green) and a background signal of the MALDI target at *m*/*z* 771.4869 (blue). (d) Overlay of ion images showing the spatial distribution of $[\text{pheophytin a+K}]^+$ at *m*/*z* 909.5291 (red),), $[\text{malonylgenistin+K}]^+$ at *m*/*z* 557.0692 (green) and a background signal of the MALDI target at *m*/*z* 771.4869 (blue). MS images were generated with 220 x 310 pixels; 20 µm pixel size; *m*/*z* bin width: $\Delta(m/z) / m/z = \pm 5$ ppm. The scale bars are (a-d) 1 mm.

Untargeted analysis of defence metabolites for wounded *Asclepias curassavica* leaf via 3D-surface MALDI MS imaging

Besides cardiac glycosides, several additional metabolites were accurately visualized, and some of them showed the same spatial distribution throughout all experiments. Up to 3513 ion signals were annotated by an unsupervised, FDR-controlled database comparison (ChEBI database, FDR: 50%). For instance, the ion signal at *m*/*z* 445.1985 was detected with a homogenous distribution in both injured areas with increased intensity after 5 hours (Figure S14a). Figure S14b shows the distribution of *m*/*z* 567.2565 that was primarily detected around the second injury (t = 10 min) indicating a short-acting defence function. However, *m*/*z* 487.1940 mainly shows a distribution around the first injury (t = 5h) (Figure S14c). The distribution pattern of *m*/*z* 621.1944 (Figure S14d) and *m*/*z* 557.2721 (Figure S14e) have high similarity with the spatial distribution of cardenolides (Figure 2d, Figure S5).The ion signal at *m*/*z* 677.1842 was primarily detected at the second injury (t = 2h) indicating that the metabolite is metabolized or delocalized after 24 hours. In total, these results are demonstrating that untargeted metabolomics in the context of MS imaging should be more widely considered as a valuable method to discover and investigate plant metabolites of potential relevance for plant interactions with herbivorous insects and pathogens.

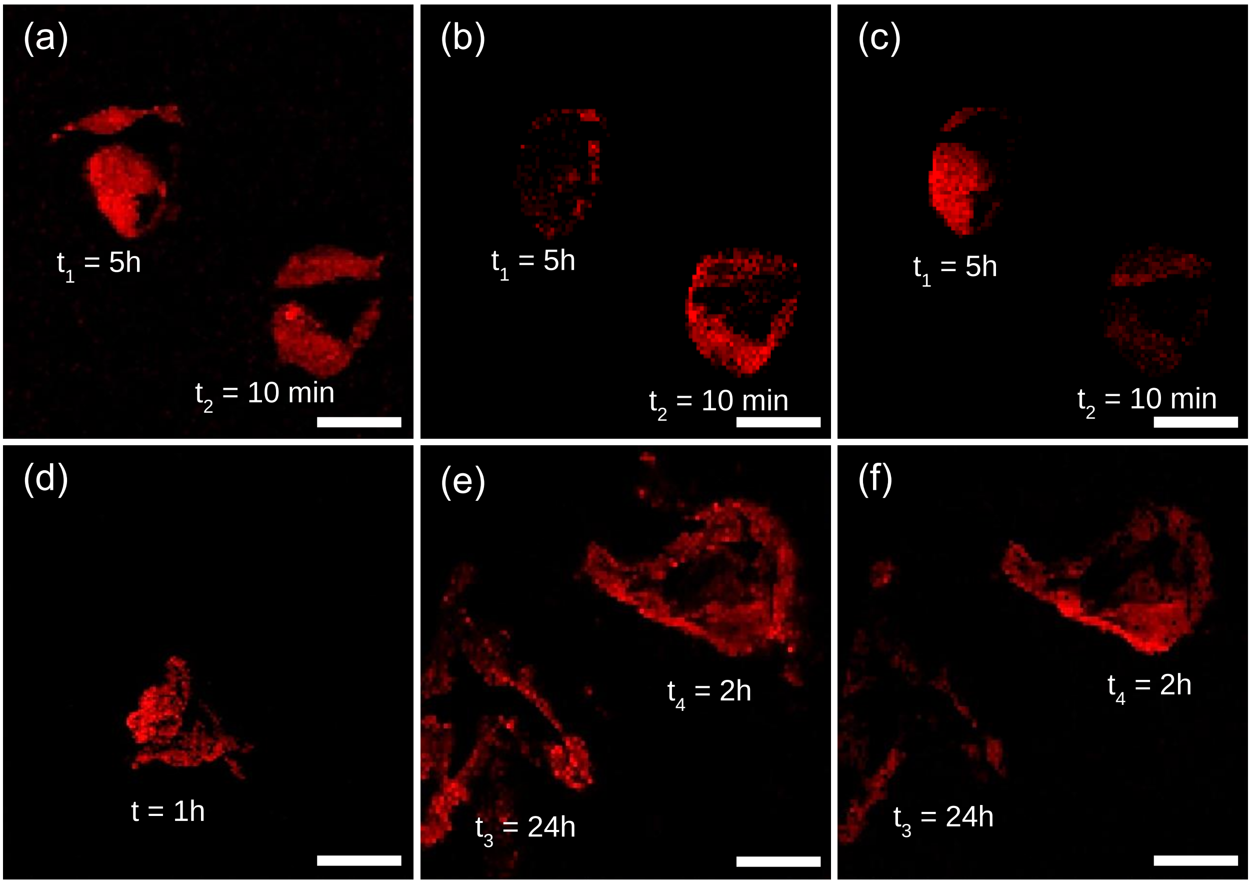

Figure S14. 3D-surface MALDI MS imaging of several Asclepias curassavica leaf samples regarding untargeted analysis for defence metabolites. (a) Ion image of *m*/*z* 445.1985. (b) Ion image of *m*/*z* 567.2565. (c) Ion image of *m*/*z* 487.1940. (d) Ion image of *m*/*z* 621.1944. (e) Ion image of *m*/*z* 557.2721. (f) Ion image of *m*/*z* 677.1842. MS images were generated with (a-c) 111 x 111 pixels, 45 µm pixel size (d) 143 x 171 pixels; 35 µm pixel size; (e,f) 112 x 103 pixels, 35 µm pixel size; (a-f) m/z bin width: Δ(m/z) / m/z = ± 5 ppm. The scale bars are 1 mm in (a-f).
